# Intrastrain Comparison of the Chemical Composition and Antioxidant Activity of an Edible Mushroom, *Pleurotus giganteus*, and Its Potent Neuritogenic Properties

**DOI:** 10.1155/2014/378651

**Published:** 2014-07-10

**Authors:** Chia-Wei Phan, Pamela David, Yee-Shin Tan, Murali Naidu, Kah-Hui Wong, Umah Rani Kuppusamy, Vikineswary Sabaratnam

**Affiliations:** ^1^Mushroom Research Centre, Institute of Biological Sciences, Faculty of Science, University of Malaya, 50603 Kuala Lumpur, Malaysia; ^2^Institute of Biological Sciences, Faculty of Science, University of Malaya, 50603 Kuala Lumpur, Malaysia; ^3^Department of Anatomy, Faculty of Medicine, University of Malaya, 50603 Kuala Lumpur, Malaysia; ^4^Department of Biomedical Science, Faculty of Medicine, University of Malaya, 50603 Kuala Lumpur, Malaysia

## Abstract

Two strains of *Pleurotus giganteus* (commercial and wild) were tested for their ability to induce neurite outgrowth in rat pheochromocytoma (PC12) and mouse neuroblastoma-2a (N2a) cells. Treatment with the mushroom extracts resulted in neuronal differentiation and neuronal elongation, but not nerve growth factor (NGF) production. Linoleic acid (4.5–5.0%, w/w) which is a major fatty acid present in the ethanol extract promoted NGF biosynthesis when augmented with low concentration of NGF (5 ng/mL). The two strains of mushroom were found to be high in protein (154–192 g kg^−1^), total polysaccharides, phenolics, and flavonoids as well as vitamins B1, B2, and B3. The total phenolics present in the mushroom extracts were positively correlated to the antioxidant activity (free radical scavenging, ferric reducing power, and lipid peroxidation inhibition). To conclude, *P. giganteus* could potentially be used in well-balanced diet and as a source of dietary antioxidant to promote neuronal health.

## 1. Introduction

Neurite outgrowth is a critical process in neuronal formation and development. Malfunction of this event will lead to demolition of synaptic connections and extended series of neuronal dysfunctions like Alzheimer's disease (AD) [1]. As AD progresses, programmed apoptotic neuronal death is triggered as a result of nerve growth factors (NGFs) depletion and oxidative stress exerted by reactive oxygen species (ROS) [2]. Therefore, strategies aimed at preserving and restoring the neurite network might be beneficial in treating AD. Dietary intake of antioxidants is also important to improve the intrinsic antioxidant mechanisms (superoxide dismutase, catalase, and glutathione peroxide) to avoid an environment where prooxidant species overwhelm antioxidant species.

Functional food plays a significant role in preventing or reducing severity of lifestyle diseases and improving physical and mental well-being of consumers. Edible and medicinal mushrooms are gaining recognition as preventative agents for age-related diseases including neurodegenerative diseases such as AD, Parkinson's disease, and dementia [3]. At the current stage, medications are prescribed for mild, moderate, to severe Alzheimer's disease to help delay or prevent behavioral symptoms, but that, too, is only for a limited time. Trials with NGF for Alzheimer's disease had gained some degree of success, but the high molecular weight of the NGF protein seems to suggest that it could not cross the blood-brain barrier [4]. Considering the limitation of the existing preventive methods, intervention strategy using mushrooms as functional food is of utmost importance. Edible and medicinal mushrooms are packed with a wide array of bioactive and nutritional components that could scavenge reactive oxygen species (ROS) and exert neuroprotective effects and promote neuritogenesis and neuroregeneration.

There are ample studies available in the literature regarding the chemical composition of different mushroom species from all over the world. However, such information on* Pleurotus giganteus* (Berk.) Karunarathna & K.D. Hyde, a popular edible mushroom in China, Thailand, and Sri Lanka is scanty. In Malaysia, the mushroom is also consumed as a delicacy by the indigenous communities. Studies on* P. giganteus* include domestication and cultivation [5], liver protection [6], and anti-*Candida* activities [7]. It has been shown that extracts of this mushroom exhibited neurite outgrowth activity in rat pheochromocytoma (PC12) and mouse neuroblastoma-2a (N2a) cells [8, 9]. In this study, the neuritogenic effects of the commercial strain of* P. giganteus* were compared to that of the wild domesticated strain. The nutritional components including vitamins, fatty acids, and amino acids as well as the phenolic, flavonoid contents, and antioxidant activities were also evaluated and compared.

## 2. Materials and Methods

### 2.1. Mushroom Species

Fresh basidiocarps of* P. giganteus* (commercial strain KLU-M 1227) were collected from Nas Agro Farm and Dong Foong Biotech. Wild* P. giganteus* (KLU-M 1228) was collected from Ayer Hitam Forest Reserve, Puchong, Malaysia. The mushroom identity (molecular fingerprinting) was authenticated by Dr. Yee-Shin Tan from Mushroom Research Centre, University of Malaya. Voucher specimens were deposited in the Herbarium of University of Malaya. Domestication of the wild strain was carried out as previously reported [8]. The substrate formulation for basidiocarp formation of both commercial and domesticated strains was similar and consisted of rubber wood sawdust, rice bran, and calcium carbonate.

### 2.2. Chemicals

Gallic acid, rutin, 2,2-diphenyl-1-picrylhydrazyl (DPPH), L-ascorbic acid, ABTS [2,2′-azino-bis-(3-ethylbenzothiazoline-6-sulfonic acid) diammonium salt], phosphate buffered saline (PBS), nerve growth factor (NGF), and dimethyl sulfoxide (DMSO) were purchased from Sigma (St. Louis, MO, USA). All other chemicals and solvents were of analytical grade and purchased from Merck (Darmstadt, Germany).

### 2.3. Proximate Analysis of Basidiocarps

The freeze-dried powder of basidiocarps of* P. giganteus* was analyzed for the nutritional components using the standard American Oil Chemists' Society (AOCS) procedures [10]. Total fat, dietary fiber, and protein content were analyzed using AOAC 989.05, AOAC 985.25, and Kjeldahl method with boric acid modification, respectively. Carbohydrates were calculated using the formula carbohydrates (g) = 100 − (protein + fat + ash). Energy was calculated using the formula energy (kcal) = 4 × (protein + carbohydrate) + 9 × (fat) [10].

### 2.4. Determination of Free Sugars, Minerals, Vitamins, Fatty Acids, and Amino Acids

Free sugars and amino acids were analyzed by high performance liquid chromatography (HPLC). Minerals were determined using inductively coupled plasma optical emission spectrometry ICP-OES following the AOAC 985.01 and 922.02, as well as American Association of Cereal Chemists (AACC 40–70) procedures. Vitamin C was determined by the AOAC 967.21 method. Preparation of methyl esters of long-chain fatty acids was carried out based on AOCS Ce-2-66 test for further analysis by gas chromatography (GC). Omega-3 and Omega-6 fatty acids were analyzed using AOCS 1d-91 methods by capillary gas-liquid chromatography (GLC).

### 2.5. Preparation of Mushroom Extracts

Crude aqueous and ethanol extracts were prepared as previously described [8]. For aqueous extract, the freeze-dried basidiocarps powder was soaked in distilled water (1 : 20, w/v) for 24 h at room temperature and 200 rpm in a shaker. After double-boiling in a water bath at 100°C, the mixture was filtered (Whatman No. 4). The filtrate was then freeze-dried and kept at −20°C prior to use. To obtain crude ethanol extract, the freeze-dried powder was soaked in 95% (v/v) ethanol at room temperature for three days. The solvent was then evaporated using a rotary evaporator (Eyela N-1000, USA) and a brownish viscous extract was obtained.

### 2.6. Determination of Total Polysaccharides

The total polysaccharide content of the aqueous and ethanol extracts was determined using the phenol-sulphuric acid method with d-glucose as a reference [11]. Briefly, 1 mL of 5% phenol was added to 1 mL of sample solution, followed by 5 mL of concentrated H_2_SO_4_. The absorbance was measured after 10 min at 497 nm.

### 2.7. Determination of Total Phenolic Contents (TPC) in Mushroom Extracts

The total phenolic contents in the mushroom extracts, expressed as gallic acid equivalents (GAEs), were determined by the Folin-Ciocalteu method [12]. Fifty microliters of sample was mixed with an equal volume of Folin-Ciocalteu phenol reagent. After 3 min, 100 *μ*L of Na_2_CO_3_ (10%) was added to the mixture. The reaction was kept in the dark for 90 min, after which the absorbance was read at 750 nm using a spectrophotometer. A calibration curve was prepared with different concentrations of gallic acid (0–100 *μ*g/mL) as standard. TPC was expressed as mg GAE/g of extract.

### 2.8. Determination of Total Flavonoids in Mushroom Extracts

Total flavonoids in the mushroom extract were estimated by using the aluminum calorimetric method as previously described [13]. Mushroom extract of 150 *μ*L was mixed with an equal volume of AlCl_3_ (2%). After 10 min, the absorbance of the supernatant was measured at 435 nm by using an ELISA microplate reader (Sunrise, Austria). The total flavonoid content of the mushroom extract was expressed as rutin equivalents in microgram per gram extract (mg RE/g extract).

### 2.9. Evaluation of Antioxidant Activity of Mushroom Extracts

#### 2.9.1. DPPH Scavenging Activity Assay

The DPPH free radical scavenging activity was determined as previously described [14]. Mushroom extracts of various concentrations (5 *μ*L) were mixed with 195 *μ*L of DPPH reagent. The reduction of the DPPH radical was determined by measuring the absorption at 517 nm using a microtiterplate reader (Tecan, Austria). The radical scavenging activity was calculated using the following equation: % radical scavenging activity = [(Abs_Blank_   −  Abs_Sample_)/Abs_Blank_  × 100%], where Abs_Sample_ is the absorbance of the sample whereas Abs_Blank_ is the absorbance of the DPPH solution. The antioxidant property of the extracts was expressed in terms of IC_50_ value, that is, the concentration to quench 50% of available DPPH content. L-ascorbic acid (0–25 *μ*M) was used as standard and butylated hydroxytoluene (BHT) was used as control.

#### 2.9.2. FRAP (Ferric Reducing Antioxidant Power) Assay

The reducing power of mushrooms extracts was determined by the ferric reducing antioxidant potential (FRAP) assay as previously described [15]. To prepare FRAP reagent, 2.5 mL of FeCl_3_ *·*6H_2_O solution (20 mM in 40 mM HCl) was mixed with 2.5 mL of 2,4,6-tri(2-pyridyl)-1,3,5-triazine (TPTZ) (10 mmol/L in 300 mmol/L acetate buffer). Then, 10 *μ*L of mushroom extract was added to 300 *μ*L of the FRAP reagent after which the absorbance of the product of the reaction between Fe^2+^ and TPTZ was measured at 593 nm against a blank for each sample. Ferrous sulfate (FeSO_4_) of concentrations 0–20 mM was used as standard and BHT as control. The FRAP value was expressed as *μ*M of FeSO_4_ equivalents/g mushroom.

#### 2.9.3. Inhibition of Lipid Peroxidation

The assay was based on the thiobarbituric acid reaction method [16]. Mushroom extracts of different concentrations (0–20 mg/mL) were mixed with 0.5 mL of egg yolk suspension and 0.5 mL of FeSO_4_. The mixture was incubated at 37°C for an hour after which 0.5 mL of 20% trichloroacetic acid (TCA) and 1 mL of 0.8% thiobarbituric acid (TBA) were added. The mixture was then heated in boiling water for 15 min and centrifuged at 3500 rpm for 20 min. The absorbance of thiobarbituric acid reactive substances (TBARS) present in the supernatant was measured at 532 nm using a microtiterplate reader (Tecan, Austria). Result was expressed as percentage inhibition of lipid peroxidation at extract concentration of 10 mg/mL. BHT was used as a control in this assay.

### 2.10. Effect of Mushroom Extracts on Neuritogenesis

#### 2.10.1. Cell Culture

Mouse neuroblastoma (N2a, CCL-131) and rat pheochromocytoma cells (adherent variant, PC-12Adh) were purchased from American Type Culture Collection (ATCC; MD, USA). N2a cells were cultured in Eagle's minimum essential medium (MEM) with L-glutamine (PAA) supplemented with 10% (v/v) heat-inactivated fetal bovine serum (PAA), 100 U/mL penicillin, and 100 *μ*g/mL streptomycin. PC12 cells were maintained in F-12K medium (Sigma) supplemented with 2.5% (v/v) heat-inactivated fetal bovine serum (PAA) and 15% (v/v) horse serum (PAA). All plates were incubated at 37°C in a humidified environment of 5% CO_2_ and 95% air. The cells were routinely passaged every 2-3 days.

#### 2.10.2. Neurite Outgrowth Assay

N2a and PC12 cells were seeded at a density of 5 × 10^3^ cells in growth medium per well in 24-well culture plates and incubated overnight. Mushroom extracts (0–50 *μ*g/mL) were added to the cells and further incubated for 3 days. For N2a cells, the cells were induced to differentiate by replacing the growth medium with 5% serum medium. Nerve growth factor (50 ng/mL) was used as a positive control. After 3 days, the cells were then examined using an inverted light microscope (Nikon Eclipse TS100). Five random fields (200–300 cells/well) were examined in each well. The number of axon-like processes, defined as extensions longer than twice the cell body diameter, was recorded. The mean number of neurite-bearing cells was quantified by scoring the total number of neurite-bearing cells over the total number of viable cells per field. At least three independent experiments were conducted.

#### 2.10.3. Measurement of NGF

The NGF level in the PC12 culture medium was performed following the ChemiKine NGF sandwich enzyme-linked immunosorbent assay (ELISA) kit procedure (Merck Millipore, Germany). After treatment, the culture medium was added into a microplate precoated with anti-mouse NGF polyclonal antibody. Anti-mouse NGF monoclonal antibody was then added. After 2 h, horseradish peroxidase- (HRP-) conjugated donkey anti-mouse IgG polyclonal antibody was added to react with TMB substrate solution. The color intensity of the sample was measured at 450 nm. The level of NGF was determined from a standard curve plotted with known concentrations of NGF.

### 2.11. Statistical Analysis

Results were expressed as mean ± standard deviation (SD) (*n* = 3). Analysis of variance (ANOVA) followed by Duncan's test was performed to test for differences between means by employing Statgraphics Plus (Statistical Graphics Corp., Herndon, VA). Correlations between total polysaccharides, TPC, and antioxidant activities were determined by Pearson's correlation coefficient (*r*) with the statistical program SPSS ver. 17.0 (SPSS Inc., Chicago, IL, USA). The statistical significance of mean differences was based on *P* value of <0.05.

## 3. Results

### 3.1. Proximate Analysis of Mushroom Basidiocarps

The proximate nutritional components of commercial* P. giganteus* (KLU-M 1227) and domesticated wild* P. giganteus* (KLU-M 1228) are shown in Table 1. The commercial strain presented a significantly higher (*P* < 0.05) carbohydrate, dietary fiber, total fat, and monosaturated fat content compared to the domesticated wild strain by 3.7, 2.7, 16, and 2.1%, respectively. Both strains showed no difference in the gross energy value and saturated fat contents. However, the wild strain presented a significantly higher (*P* < 0.05) crude protein and polyunsaturated fat content.

### 3.2. Determination of Sugars, Minerals, and Vitamins

The sugar composition of* P. giganteus* basidiocarps is given in Table 1. Glucose and fructose were detected in the basidiocarps of both strains. The glucose and fructose content of the domesticated wild strain was approximately 47% and 26% higher (*P* < 0.05) than that of the commercial strain. For macroelements composition, potassium in the basidiocarps of commercial strain (13.46 ± 0.0 g kg^−1^) was significantly higher (*P* < 0.05) than the domesticated wild strain (11.71 ± 0.32 g kg^−1^). On the other hand, the calcium level in the domesticated wild strain (0.087 ± 0.01 g kg^−1^) was higher than that of the commercial strain (0.058 ± 0.0 g kg^−1^). The vitamin profiles showed similarity, but the concentrations in the two strains were different. Vitamin C (ascorbic acid) in the wild strain was almost 3.9-fold higher than that of the commercial strain (Table 1). Vitamin B3 (niacin) is the most abundant vitamin found in this mushroom with 0.09 ± 0.10 and 0.06 ± 0.02 g kg^−1^ in the commercial and domesticated wild strain, respectively.

### 3.3. Determination of Amino Acids and Fatty Acids

All the essential amino acids (threonine, valine, methionine, isoleucine, leucine, phenylalanine, tryptophan, lysine, histidine, and arginine) were detected in this mushroom with the exception of tryptophan (Table 2). Among the essential amino acids, leucine (commercial strain 20.4 ± 0.10 g kg^−1^; domesticated wild strain 19.4 ± 0.0 g kg^−1^) was found to be the largest constituent, followed by phenylalanine and histidine. The methionine, isoleucine, and valine were present in small amounts ranging from 0.31 to 0.80% for both the* P. giganteus* strains. In particular,* P. giganteus* had the highest concentration of glycine, which is categorized as the nonessential amino acid. Also, the wild strains exhibited higher amount of glutamine, alanine, and asparagine as compared to the commercial strains.

Table 3 presents the fatty acid profile of basidiocarps of* P. giganteus*. Among the saturated fatty acids, palmitic acid (C16:0) was predominant with 3.8 ± 0.01 and 3.3 ± 0.0 g kg^−1^ in the commercial strain and wild strain, respectively. Amongst the polyunsaturated fatty acids, oleic acid was the predominant fatty acid in this species. Oleic acid (C18: 1n9c) in the commercial strain (10.3 ± 0.06 g kg^−1^) was significantly (*P* < 0.05) higher than that in the wild strain (8.9 ± 0.0 g kg^−1^). Linoleic acid was also present in significant quantities (4.5–5.0 g kg^−1^) in* P. giganteus*. The medium chain fatty acid, caprylic acid (C8:0), and the long chain fatty acid, stearic acid (C18:0), were also present. Capric, undecanoic, palmitoleic, and eicosadienoic acids were detected in trace amounts in* P. giganteus.*

### 3.4. Determination of Total Polysaccharides, Total Phenolic, and Total Flavonoids Compound

The total polysaccharides of the commercial and wild* P. giganteus* were comparable. The aqueous extract had the highest amount of polysaccharides as shown in Table 4. Notably, the total polysaccharides in aqueous extract of the wild strains (17.91 ± 0.05%, w/w) were significantly (*P* > 0.05) higher than those of the commercial strain (14.93 ± 0.04%). On the other hand, the total phenolic content in the ethanol extracts was higher. The quantity of phenolics in the mushrooms extracts was in descending order: commercial strain ethanol extract > wild strain ethanol extract > commercial extract aqueous extract > wild strain aqueous extract. There was also a significant difference (*P* < 0.05) in the total flavonoids between the basidiocarps of the commercial and wild strains. The wild* P. giganteus* had approximately 2.09% higher flavonoids than the commercial strains.

### 3.5. Evaluation of Antioxidant Activity of Mushroom Extracts

#### 3.5.1. DPPH Scavenging Activity Assay

The scavenging effect of the mushroom extracts on DPPH radicals increased with sample concentration, depending on the extraction solvent and strain type. In general, the ethanol extracts showed higher scavenging activity, hence lower IC_50_ (mg/mL) when compared to the aqueous extracts (Table 5). The scavenging activity obtained in descending order was wild strain ethanol extract > commercial strain ethanol extract > wild strain aqueous extract > commercial strain aqueous extract.

#### 3.5.2. FRAP (Ferric Reducing Antioxidant Power) Assay

In the ferric reducing power assay, the reducers present in the mushroom extracts lead to the reduction of the Fe^3+^/ferricyanide complex to the ferrous form. The reducing capacity of the mushroom extract serves as an index of antioxidant activity. The reducing ability of the different extracts was in the range of 1.17–3.88 *μ*M FeSO_4_ *·*7H_2_O/g mushroom (Table 5). The antioxidant activity obtained in descending order was commercial strain ethanol extract ≥ wild strain ethanol extract ≥ commercial strain aqueous extract ≥ commercial strain aqueous extract.

#### 3.5.3. Inhibition of Lipid Peroxidation

The study of lipid peroxidation (LPO) inhibition is based on the measurement of malondialdehyde (MDA) generated by the polyunsaturated fatty acid peroxides upon decomposition. As a result of LPO, destruction of cellular components occurs and brings about oxidative stress in biological systems. As shown in Table 5, there was no significant difference (*P* > 0.05) in terms of lipid peroxidation inhibition between the commercial and domesticated wild mushroom extracts. However, the ethanol extracts of both the strains showed significantly higher (*P* < 0.05) lipid peroxidation inhibitory ability (49.58–49.80%) when compared to the aqueous extracts (44.41–44.61%). Therefore, the means of extractions instead of mushroom strains played a more prevailing role in lipid peroxidation inhibition.

### 3.6. Correlation between Total Polysaccharides, TPC, and Antioxidant Parameters

The TPC in the mushroom extract was positively correlated to the DPPH scavenging capacity (*r* = +.827) and FRAP reducing power (*r* = +.820). This indicated that the antioxidant effects increased with increasing concentrations of the total phenolics present in the mushroom extracts. However, a weak correlation was found between the lipid peroxidation inhibition activity and the TPC (*r* = +.321). Nevertheless, the DPPH scavenging capacity, FRAP reducing power, and lipid peroxidation inhibition showed a strong positive correlation (*r* = +.806 to +.820) between each antioxidant activity.

### 3.7. Neurite Outgrowth Assay

**Figure 1:**
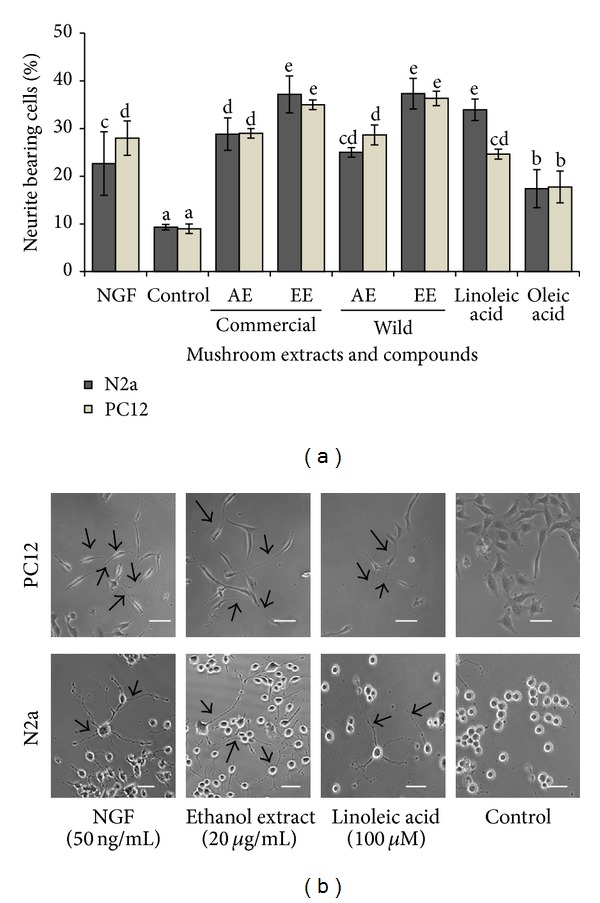
(a) Neurite-bearing cells (%) after 3-day incubation with NGF, mushroom extracts, linoleic acid, and oleic acid. AE: aqueous extract; EE: ethanol extract. Data are expressed as mean ± SD of three experiments. Different letters represent significant differences between samples (*P* < 0.05). (b) Phase-contrast photomicrographs showing the effects of NGF, ethanol extract, and linoleic acid on the morphology of PC12 and differentiating N2a cells after 3 days. Untreated cells serve as control and only contained 5% FBS as vehicle. Arrows indicate neurite extension and scale bar corresponds to 20 *μ*m.

The mean value of neurite-bearing cells in NGF treated cells (positive control) was 22.67 ± 6.67% as shown in Figure 1(a). The ethanol extracts of the commercial and wild* P. giganteus* (20 *μ*g/mL) caused a significant (*P* < 0.05) increase in neurite-bearing cells by 3.98- and 4-fold, respectively, when compared to the control cells with medium only. Additionally, in order to verify the neuritogenic activity of these mushroom extracts, PC12 cells, which only extend neurite upon NGF activation, were employed. Incubation of PC12 with 20 *μ*g/mL of ethanol extracts resulted in a significant increase (*P* < 0.05) in neurite-bearing cells compared to cells treated with NGF alone. Phase-contrast micrographs of neurite-bearing cells were shown in Figure 1(b). The two most abundant fatty acids in the investigated extract were oleic acid and linoleic acid (Table 3). Therefore, the neuronal cells were treated with linoleic acid and oleic acid and the neurite outgrowth activity was examined. Linoleic acid enhanced the neuritogenic activity of PC12 and N2a cells significantly (*P* < 0.05) but not oleic acid (Figure 1(a)).

### 3.8. NGF Measurement

**Figure 2:**
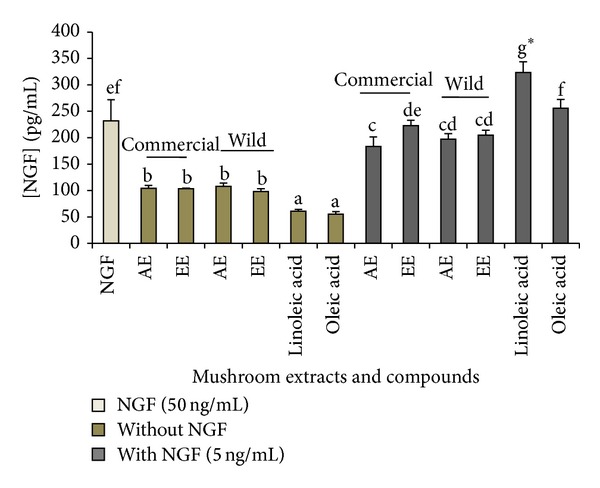
The effects of mushroom aqueous and ethanol extracts on the stimulation of NGF secretion by neurite-bearing PC12 cells in the presence or absence of NGF. NGF (50 ng/mL) was used as the positive control. Values are mean ± SD from three independent experiments. Different letters represent significant differences between samples (*P* < 0.05). *P < 0.05 represents a significant difference from the control, that is, 50 ng/mL of NGF.

We measured the level of NGF in the culture medium after PC12 cells were cultivated in the presence of* P. giganteus* extracts and linoleic and oleic acids for 3 days. Linoleic acid significantly (*P* < 0.05) augmented NGF secretion by 1.4 times (Figure 2). On the other hand, oleic acid had a lower potency in promoting NGF secretion (255 pg/mL), as compared to linoleic acid (323 pg/mL). In contrast to the fatty acids, the extracts had a weak stimulatory effect on NGF secretion.

## 4. Discussion

The nutritional components found in the present work are in accordance with the literature. A study in Italy showed that the protein content in* Pleurotus ostreatus* (grey oyster mushroom) was 1.61 ± 0.02 g/100 g, which is lower than the protein content of* P. giganteus* in this study [17].* Pleurotus giganteus* also showed a higher protein content when compared to* Pleurotus sajor-caju* (13.0–18.4 g/100 g) [18]. The protein content of mushrooms is dependent on the strain, substrate chemical composition, pileus size, and cultivation time [19, 20]. In this study, rice bran supplementation may have increased the soluble protein content present in both strains as the protein content in rice bran is about 10–15% of the total weight of the mushroom growth substrate [21].

*Pleurotus ostreatus* was composed mainly of glucose (14.29 g kg^−1^) and mannose (10.55 g kg^−1^) [22]. While fructose was not detected in* P. ostreatus*, 27.81 g kg^−1^ of glucose was detected in* P. eryngii* with trace amounts of ribose and xylose.* Pleurotus* spp. in particular are rich in calcium, potassium, magnesium, iron, and phosphorus [23].* Pleurotus sajor-caju*,* P. platypus*, and* P. citrinopileatus* were reported to contain 16.3 ± 0.22, 11.2 ± 0.3, and 10.3 ± 0.2 g kg^−1^ of potassium, respectively. The concentrations of vitamin B3 in mushrooms were highly species dependent and they vary from 34 to 109 mg/100 g dry weight for* P. ostreatus* [24]. Mushrooms contain higher vitamin B2 (riboflavin) as compared to vegetables. Some varieties of* Agaricus bisporus* (white button mushroom) have also been reported to contain vitamin B2 levels as high as those found in eggs and cheese [25]. In our study, vitamin B1 (thiamin) was higher in the commercial strain by 1.9-fold, while vitamin B2 level was not significantly different when compared to that of the wild strain.

The free amino acids in* P. giganteus* were comparable with reported literature values. Tanzanian wild mushrooms* Lactarius* sp. (milky cap),* Boletus pruinatus* (Matt Bolete mushroom), and* Boletinus cavipes* were reported to have leucine as high as 15.9%, 10.6%, and 8.40%, respectively [26]. The fresh* P. ostreatus* was found to have 2.74, 1.76, and 1.43 g kg^−1^ of glutamine, asparagine, and arginine, respectively [27]. Similar to our findings, other edible mushrooms also contained high levels of oleic acid (% total fatty acid methyl esters):* Auricularia polytricha* (27.1),* Lentinus sajor-caju* (23.5),* Lentinus squarrosulus* (5.8),* Pleurotus djamor* (28.8),* P. sajor-caju* (16.4), and* Russula brevipes *(39.2) [28]. The presence of caprylic and capric acids in mushrooms is rare.* Calocybe gambosa* (St. George's mushroom) and* Clitocybe odora* (Aniseed funnel mushroom) were reported to have 0.25 ± 0.02 and 0.03 ± 0.00% of caprylic acid, respectively, while* Coprinus comatus* (shaggy mane mushroom) registered 0.09 ± 0.00% of capric acid [29].

Extraction with boiling water is used to obtain extracts with high molecular weight compounds, such as polysaccharides which play an important medicinal role in mushrooms [30]. Low molecular weight compounds, such as phenolic compounds, were usually from ethanol extraction [29]. Although flavonoid was not detected in* P. sajor-caju* [31], its concentration ranging from 0.24 to 0.32 mg/g was reported in* P. ostreatus* [32]. Flavonoids were also present in other mushroom species like* Clitocybe gibba* (3.56 mg chatequin/g extract) and* Boletus armeniacus* (8.59 mg chatequin/g extract) [33]. The rubber wood sawdust which is rich in lignin could have been depolymerized by the lignocellulosic enzymes of mushrooms into phenolic units and further dimerized or polymerized, creating flavonoids [34].

DPPH radical scavenging effects of the mycelia extracts of* Pleurotus* spp. (*P. citrinopileatus*,* P. djamor*,* P. eryngii*,* P. flabellatus*,* P. florida*,* P. ostreatus*, and* P. sajor-caju*) have been reported [32, 35]. The methanol and hot water extracts (10 mg/mL) of* P. eous* were found to scavenge DPPH radical by 85.19% and 70.21%, respectively [36]. The higher DPPH scavenging ability of ethanol extracts might be due to more hydrogen-donating components including phenolic compounds extracted from the mushroom. The higher phenolic content of ethanol extracts might account for the better results found in reducing power as compared to the aqueous extract [37]. When comparing the FRAP values of* P. giganteus* with* P. sajor-caju*, FRAP values of aqueous extract of* P. giganteus* were lower than those of* P. sajor-caju* (35.06 ± 0.86 *μ*M FeSO_4_ *·*7H_2_O/g mushroom) [31]. The higher phenolic content in the ethanol extracts might contribute to a higher inhibitory effect on lipid peroxidation.* Pleurotus florida* was reported to display 57% of lipid peroxidation inhibition, while* P. flabellatus*,* P. cystidiosus*,* P. eryngii*, and* P. sajor-caju* showed 50%, 49.8%, 48%, and 43% of lipid peroxidation inhibition, respectively [38]. Meanwhile,* P. ostreatus* at a concentration of 10 mg/mL inhibited LPO activity in rat liver homogenate by 56.20% [39]. In accordance with the present results, Dubost et al. [34] had demonstrated a positive correlation between the TPC in the mushroom extracts of* P. ostreatus* and* P. eryngii* and the antioxidant capacity.

It has been reported that tiger's milk mushroom,* Lignosus rhinocerotis*, demonstrated neuritogenic effects on PC12 cells [40]. The aqueous extract of* Ganoderma neo-japonicum* (50 *μ*g/mL) was found to trigger a maximal stimulation of PC12 neurite outgrowth with 14.22 ± 0.43% of neurite-bearing cells [41]. Extension of neurites from neuronal cell body is an important step in neuronal development and requires the generation of additional plasma membrane [42]. Polyunsaturated fatty acids like linoleic, linolenic, docosahexanoic, and arachidonic acids promoted basal and nerve growth factor- (NGF-) induced neurite extension of the PC12. This suggested that linoleic acid which was present in abundance in the extracts of* P. giganteus* may play a key role in neuritogenesis. In contrast, studies showed that monounsaturated fatty acids and saturated long-chain fatty acids like oleic, stearic, and palmitic acids caused little or no effects in neurite outgrowth [43]. Studies have shown that cyathane diterpenes from the mushroom* Sarcodon cyrneus*, namely, cyrneines A, B, C, and D, and glaucopine C increased the NGF gene expression in 1321N1 astrocytoma cells [44]. Hericenones C, D, and E isolated from the basidiocarps of* Hericium erinaceus* exhibited stimulating activity for the biosynthesis of NGF* in vitro*. In the presence of hericenones C, D, and E at 33 *μ*g/mL, mouse astroglial cells secreted 10.8 ± 0.8, 23.5 ± 1.0, 13.9 ± 2.1, and 45.1 ± 1.1 pg/mL of NGF into the culture medium, respectively [45].

Long-chain polyunsaturated fatty acids (LCPUFA) are essential nutrients in the development and functioning of the brain and central nervous system. The most abundant LCPUFA in the brain are docosahexaenoic acid (DHA) which is mainly derived from fish and arachidonic acid (ARA) from meat and eggs [46]. The desaturation and elongation of linoleic acids and alpha-linolenic acids to ARA and DHA, respectively, are very crucial for the infant's brain development. Since the percentage of ARA decreases in the brain during prenatal development, the balance in the dietary ratio of linoleic acid is very crucial to the brain development in preterm infant [47]. Apart from that, many studies have demonstrated the importance of LCPUFA as potent neuroprotectant. Linoleic acid was found to protect mouse cortical neurons against glutamate excitotoxicity [48]. Linoleic acid and its derivatives also prevented sodium nitroprusside-induced cell death of cultured rat cerebral cortical neurons [49]. However, to date, limited information is available on the role of polyunsaturated fatty acid as a stimulator of NGF synthesis in neuronal cells.

## 5. Conclusion

In conclusion, we report for the first time the chemical compositions of the commercial and domesticated wild strains of* P. giganteus*. The extracts of both the strains showed distinctive antioxidant activities due to the differential distributions of total phenolics and flavonoids. Aqueous extracts did not contain flavonoids and had a lower phenolic content, hence explaining its lower antioxidative capacity. Our study demonstrates that the ethanol extract and its major constituent, linoleic acid, induced neurite outgrowth and increased NGF biosynthesis. These findings provide support for the possible role of* P. giganteus* as a functional food to maintain neuronal differentiation and neuritogenesis, as well as a healthy NGF supply in central and peripheral nervous system.

## Figures and Tables

**Table 1:** Chemical compositions, sugars, macro- and microelements, and vitamins of commercial *Pleurotus giganteus* (KLU-M 1227) and wild *P. giganteus* (KLU-M 1228).

Parameter	*Pleurotus giganteus* (commercial strain)	*Pleurotus giganteus* (wild strain)
Carbohydrate (g kg^−1^)	672 ± 0.0^a^	647 ± 0.0^b^
Protein (g kg^−1^)	154 ± 0.0^c^	192 ± 0.0^d^
Energy (kcal kg^−1^)	3640 ± 0.00^e^	3640 ± 0.0^e^
Dietary fiber (g kg^−1^)	333.5 ± 0.07^f^	324.5 ± 0.07^g^
Total fat (g kg^−1^)	37.0 ± 0.0^h^	31.0 ± 0.0^i^
Saturated fat	9.7 ± 0.0^j^	9.5 ± 0.0^j^
Monosaturated fat	19.7 ± 0.0^k^	13.2 ± 0.0^l^
Polyunsaturated fat	7.8 ± 0.0^m^	8.3 ± 0.0^n^
Trans fat	ND	ND
Cholesterol (g kg^−1^)	ND	ND
Free sugars (g kg^−1^)		
Fructose	7.2 ± 0.2^a^	10.6 ± 0.4^b^
Glucose	31.4 ± 0.7^c^	39.7 ± 0.2^d^
Sucrose	ND	ND
Lactose	ND	ND
Maltose	ND	ND
Maltotriose	ND	ND
Macroelements (g kg^−1^)		
Potassium (as K)	13.46 ± 0.0^a^	11.71 ± 0.32^b^
Phosphorus (as P)	5.27 ± 0.39^c^	4.01 ± 0.04^d^
Magnesium (as Mg)	0.67 ± 0.00^e^	0.65 ± 0.00^f^
Calcium (as Ca)	0.058 ± 0.00^g^	0.087 ± 0.01^h^
Sodium (as Na)	0.058 ± 0.07^g^	0.047 ± 0.00^i^
Microelements (g kg^−1^)		
Iron (as Fe)	0.019 ± 0.04^a^	0.014 ± 0.00^b^
Zinc (as Zn)	0.027 ± 0.01^c^	0.042 ± 0.00^d^
Manganese (as Mn)	0.041 ± 0.01^e^	0.043 ± 0.00^e^
Copper (as Cu) (mg kg^−1^)	0.60 ± 0.01^f^	0.228 ± 0.00^f^
Selenium (as Se) (mg kg^−1^)	ND	ND
Vitamins (g kg^−1^)		
Vitamin B1	0.004 ± 0.01^a^	0.002 ± 0.00^b^
Vitamin B2	0.009 ± 0.00^c^	0.009 ± 0.00^c^
Vitamin B3	0.09 ± 0.10^d^	0.06 ± 0.02^e^
Vitamin C	0.003 ± 0.00^a^	0.001 ± 0.01^f^

Each value is expressed as mean ± SD (*n* = 3). In each row, the different letters represent significant differences between samples (*P* < 0.05). ND: not detectable.

**Table 2:** Amino acid content (g kg^−1^protein) of *Pleurotus giganteus* KLU-M 1227and KLU-M 1228.

Amino acids	*Pleurotus giganteus* (commercial strain)	*Pleurotus giganteus* (wild strain)
Asp	6.9 ± 0.0^c^	7.0 ± 0.0^c^
Glu	15.7 ± 0.0^jk^	17.2 ± 0.1^l^
Ser	11.1 ± 0.01^e^	11.0 ± 0.01^e^
His∗	15.4 ± 0.04^ji^	15.1 ± 0.05^j^
Gly	29.0 ± 0.11^p^	29.5 ± 0.04^p^
Thr∗	5.3 ± 0.01^b^	5.2 ± 0.00^b^
Arg∗	11.4 ± 0.04^ef^	12.1 ± 0.00^fg^
Ala	11.1 ± 0.01^e^	13.5 ± 0.00^h^
Tyr	5.8 ± 0.06^b^	5.9 ± 0.00^b^
Cys	ND	ND
Val∗	8.0 ± 0.01^d^	7.1 ± 0.00^c^
Met∗	3.1 ± 0.01^a^	3.1 ± 0.00^a^
Phe∗	16.4 ± 0.09^kl^	16.5 ± 0.00^kl^
Ile∗	7.7 ± 0.02^cd^	7.3 ± 0.00^cd^
Leu∗	20.4 ± 0.10^n^	19.4 ± 0.00^m^
Lys∗	14.5 ± 0.01^i^	12.8 ± 0.00^gh^
Asn	15.2 ± 0.07^ij^	24.6 ± 0.00^o^
Gln	ND	ND
Nva	ND	ND
Trp∗	ND	ND

Asp: aspartate; Glu: glutamate; Ser: serine; His: histidine; Gly: glycine; Thr: threonine; Arg: arginine; Ala: alanine; Tyr: tyrosine; Cys: cystine; Val: valine; Met: methionine; Phe: phenylalanine; Ile: isoleucine; Leu: leucine; Lys: lysine; Asn: asparagine; Gln: glutamine; Nva: norvaline; Trp: tryptophan. Each value is expressed as mean ± SD (*n* = 3). In each row, the different letters represent significant differences between samples (*P* < 0.05). ND: not detected. ∗Essential amino acids.

**Table 3:** Fatty acids (g kg^−1^dry weight) detected in the basidiocarps of *Pleurotus giganteus*.

	Fatty acid	*Pleurotus giganteus* (commercial strain)	*Pleurotus giganteus* (wild strain)
C8:0	Caprylic	1.3 ± 0.0^a^	1.1 ± 0.0^ab^
C10:0	Capric	0.4 ± 0.0^c^	0.4 ± 0.0^c^
C11:0	Undecanoic	0.8 ± 0.0^b^	0.8 ± 0.0^b^
C16:0	Palmitic	3.8 ± 0.0^d^	3.3 ± 0.0^e^
C16:1	Palmitoleic	0.1 ± 0.0^c^	0.1 ± 0.0^c^
C18:0	Strearic	1.1 ± 0.03^ab^	0.8 ± 0.0^b^
C18:1n9c	Oleic∗∗∗	10.3 ± 0.06^g^	8.9 ± 0.0^h^
C18:2n6c	Linoleic∗∗	5.0 ± 0.01^f^	4.5 ± 0.0^f^
C20:2	Eicosadienoic∗∗	0.3 ± 0.01^c^	0.3 ± 0.0^c^
C22:0	Beheric	0.1 ± 0.0^c^	0.1 ± 0.0^c^
C24:0	Lignoceric	0.4 ± 0.01^c^	0.4 ± 0.0^c^

∗∗Omega-6 PUFAs; ∗∗∗Omega-9 PUFAs. Each value is expressed as mean ± SD (*n* = 3). In each row, the different letters represent significant differences between samples (*P* < 0.05).

**Table 4:** Total polysaccharides, phenolics, and flavonoids present in the crude aqueous and ethanol extracts of basidiocarps of *Pleurotus giganteus*.

	*Pleurotus giganteus* (commercial strain)		*Pleurotus giganteus* (wild strain)	
	Aqueous	Ethanol	Aqueous	Ethanol
Extraction yield (%, w/w)	15.60 ± 2.20^a^	12.00 ± 1.00^b^	13.77 ± 1.68^ab^	6.67 ± 1.06^c^
Total polysaccharides (%, w/w)	14.93 ± 0.04^a^	11.31 ± 0.16^b^	17.91 ± 0.05^c^	13.72 ± 0.04^d^
Total phenolic content (mg GAE/g)	12.14 ± 1.89^a^	24.08 ± 1.04^b^	9.58 ± 0.18^c^	21.61 ± 1.47^d^
Total flavonoids (mg RE/g)	ND	2.94 ± 0.00^a^	ND	6.14 ± 0.01^b^

In each row, the different letters represent significant differences between samples (*P* < 0.05). ND = not detected.

**Table 5:** Antioxidant activities of the aqueous and ethanol extracts from the commercial strain of *Pleurotus giganteus* (KLU-M 1227) and the wild strain (KLU-M 1228).

Antioxidant properties	Test method	Positive control (BHT)	*Pleurotus giganteus *		*Pleurotus giganteus *	
			(commercial strain)		(wild strain)	
			Aqueous	Ethanol	Aqueous	Ethanol
Free radical scavenging	DPPH (IC_50_; mg/mL)	0.09 ± 0.01	21.46 ± 6.95^a^	11.28 ± 3.54^bc^	16.18 ± 1.76^ab^	8.10 ± 2.15^c^
Reducing power	FRAP (*µ*M FeSO_4_ *·*7H_2_O/ g)	780.29 ± 13.4	2.26 ± 0.29^ab^	2.99 ± 0.14^b^	2.04 ± 0.32^a^	2.69 ± 0.71^b^
Lipid peroxidation inhibition	Inhibition of lipid peroxidation at extract concentration of 10 mg/mL (%)	79.07 ± 2.25	44.41 ± 1.00^a^	49.58 ± 1.87^b^	44.61 ± 1.42^a^	49.80 ± 3.27^b^

In each row, the different letters represent significant differences between samples (*P* < 0.05).
